# Antiquity of the Trephine

**Published:** 1884-02

**Authors:** 


### The Antiquity of the Trephine.

In an editorial on this subject in the *Medical News*, the following occurs:

The trephine dates back to at least five hundred years before Christ. No such instrument, however, existed in the Stone Age, and the openings are of such character as to forbid the surmise that any similar instrument was used. Most probably it was done by scraping, a method which, while tedious and painful in the adult, Broca has shown would be very brief, not exceeding four minutes, in the thinner and softer skull of childhood.

